# Early bile drainage improves native liver survival in biliary atresia without cholangitis

**DOI:** 10.3389/fped.2023.1189792

**Published:** 2023-07-12

**Authors:** Fei Liu, Xiaogang Xu, Zijian Liang, Boyuan Tao, Menglong Lan, Jixiao Zeng

**Affiliations:** Department of Pediatric Surgery, Guangzhou Women and Children's Medical Center, Guangzhou Medical University, Guangdong Provincial Clinical Research Center for Child Health, Guangzhou, China

**Keywords:** biliary atresia, cholangitis, portoenterostomy, native liver survival, jaundice clearance

## Abstract

**Objectives:**

To explore the outcomes and related factors in children without cholangitis after Kasai portoenterostomy (KPE).

**Methods:**

We retrospectively analyzed the data of infants with type III BA who underwent KPE from June 2016 to December 2021. We compared and analyzed the difference in native liver survival (NLS) rates in different types of cholangitis. We also investigated the relationship between the absence of cholangitis and the effect of early bile drainage (EBD) as well as the related factors affecting EBD efficacy.

**Results:**

A total of 145 children were included in this study. Among these children, 82 (56.6%, 82/145) had cholangitis, including 40 (48.8%, 40/82) with early cholangitis and 33 (40.2%, 33/82) with recurrent cholangitis. The median follow-up period was 29 months (range, 2–75 months). The NLS rates were 67.6%, 51.7%, 45.5% and 43.4% at 6 months, 1 year, 2 years and 5 years following KPE, while the NLS rates for infants without cholangitis after KPE were 68.3%, 50.8%, 46.0% and 46.0%, respectively. Higher gamma-glutamyl transferase (γ- GT) and total bile acid (TBA) before KPE were risk factors for cholangitis (*P *< 0.05). The NLS rate in recurrent cholangitis was significantly lower than that in occasional cholangitis (*P *< 0.01). Compared with the EBD-poor group, the NLS rate in the EBD-good group of infants was significantly increased (*P *< 0.001). EBD was significantly correlated with the occurrence and frequency of cholangitis (*P *< 0.05).

**Conclusions:**

Recurrent cholangitis was an important factor affecting NLS. For children without cholangitis after KPE, early bile drainage was better, and the NLS was longer.

## 1. Introduction

Biliary atresia (BA) is a serious life-threatening neonatal obstructive jaundice disease that is characterized by progressive inflammation of intrahepatic and extrahepatic bile ducts and eventually develops into cirrhosis and liver failure. If left untreated, most patients will die before the age of 2 (1). Although Kasai portoenterostomy (KPE) is the primary treatment for BA, nearly 70% of children ultimately need liver transplantation (LT), even after successful KPE (2–4).

Cholangitis is the most common complication of BA after KPE and is also an important factor that seriously affects native liver survival (NLS) (5). The occurrence of cholangitis may be related to gut microbiota migration and the immune inflammatory response (6–8). Postoperative cholangitis, especially recurrent cholangitis, can significantly reduce the NLS rate in children with BA (9, 10). Most studies have focused on the relationship between cholangitis and NLS, and there are few studies on the relationship between the absence of cholangitis after KPE and NLS. The aims of this study were (1) to explore the relationship between cholangitis and NLS and its related factors and (2) to focus on the outcome and related factors of children without cholangitis after KPE.

## 2. Methods

### 2.1. Patient information

This study retrospectively analyzed the data of 145 infants with type III BA who underwent KPE in the Guangzhou Women and Children's Medical Center from June 2016 to December 2021. All the children underwent KPE performed by the same surgeons. The inclusion criteria of this study included the following: (1) All patients were diagnosed with BA by laparoscopic biliary exploration and postoperative pathology and were classified as type III (11). (2) The patients and their families cooperated with the doctor for treatment, took regular medication after surgery, and be reviewed regularly as needed, with complete follow-up data. The exclusion criteria included patients that did not complete the follow-up, cases with data loss and cases of research refusal. Informed written consent was acquired from the parents or guardians of infants in the study. The protocol was approved by the Ethics Committee of Guangzhou Women and Children's Medical Center (approval no. 486B00), and the research was conducted in compliance with the World Medical Association Declaration of Helsinki.

### 2.2. Definition of cholangitis and staging of liver fibrosis

Cholangitis was defined as the presence of systemic inflammation (fever ≥38.0°C or elevated inflammatory markers, such as the white blood cell [WBC] count and C-reactive protein [CRP] level), with evidence of cholestasis (jaundice aggravates or regresses, stool color becomes lighter or acholic stool reappears, serum total bilirubin and direct bilirubin increases) (11, 12). To better explore the relationship between cholangitis and NLS, cholangitis occurring within 1 month after KPE was defined as early cholangitis, and cholangitis occurring 1 month after KPE was defined as late cholangitis (6, 13). A frequency of cholangitis ≤3 times within 6 months after KPE was considered to be occasional cholangitis, and a frequency of >3 times was considered to be recurrent cholangitis. To better investigate the NLS of children without cholangitis after KPE, “early bile drainage-good (EBD-G)” was introduced and defined as the color of stool turning yellow, yellow staining of the skin and sclera obviously subsiding and serum bilirubin level decreasing by 1/3 of that before the operation 1 month after KPE. Conversely, it was defined as “early bile drainage-poor (EBD-P).” This study used the Metavir score to stage liver fibrosis, with the following scores: F0 (no fibrosis), F1 (fibrous portal expansion), F2 (few bridges or septa), F3 (numerous bridges or septa) and F4 (cirrhosis) (1, 14).

### 2.3. Follow-up

Postoperative follow-ups were conducted for all patients at 1, 2, 3 and 6 months after surgery completion and every 6 months thereafter. Each visit consisted of a physical examination, abdominal ultrasound and liver function tests. Telephone follow-up was conducted every six months after surgery. The end point of follow-up was death or liver transplantation, and the follow-ups occurred until September 2022.

### 2.4. Statistical analysis

SPSS 21.0 software was used for statistical analysis. Student's *t*-test was used for comparisons between two groups with measurement data, and the *χ*^2^ test or Fisher's exact test was used for categorical variables. The Mann‒Whitney *U* test was used for grade data. A Cox regression test was used to compare the NLS rate. A value of *P *< 0.05 indicated that the difference was statistically significant.

## 3. Results

### 3.1. Characteristics of the study population

**Figure 1:**
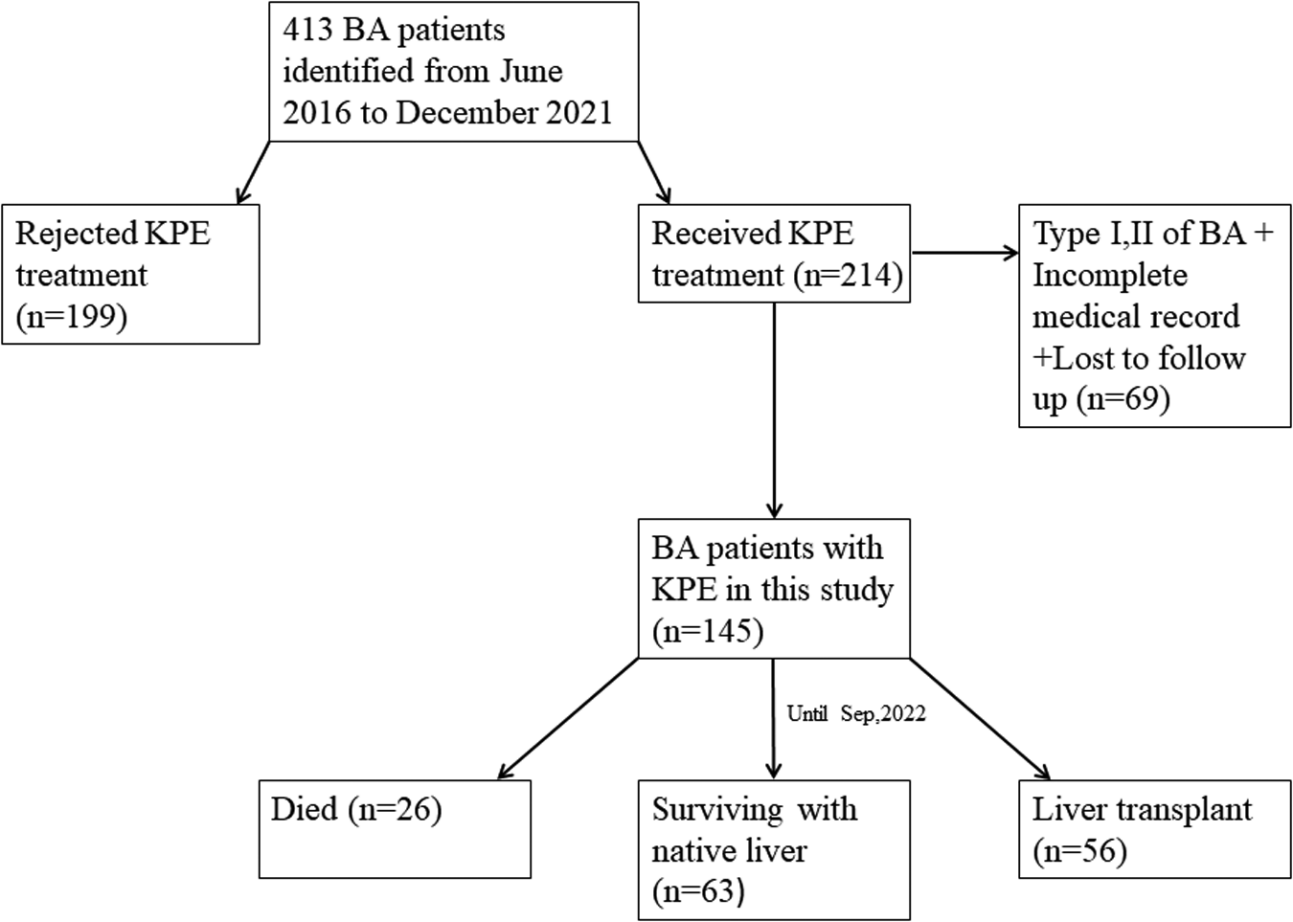
Flowchart of patients recruitment in this study. BA, biliary atresia; KPE, Kasai portoenterostomy.

**Figure 2:**
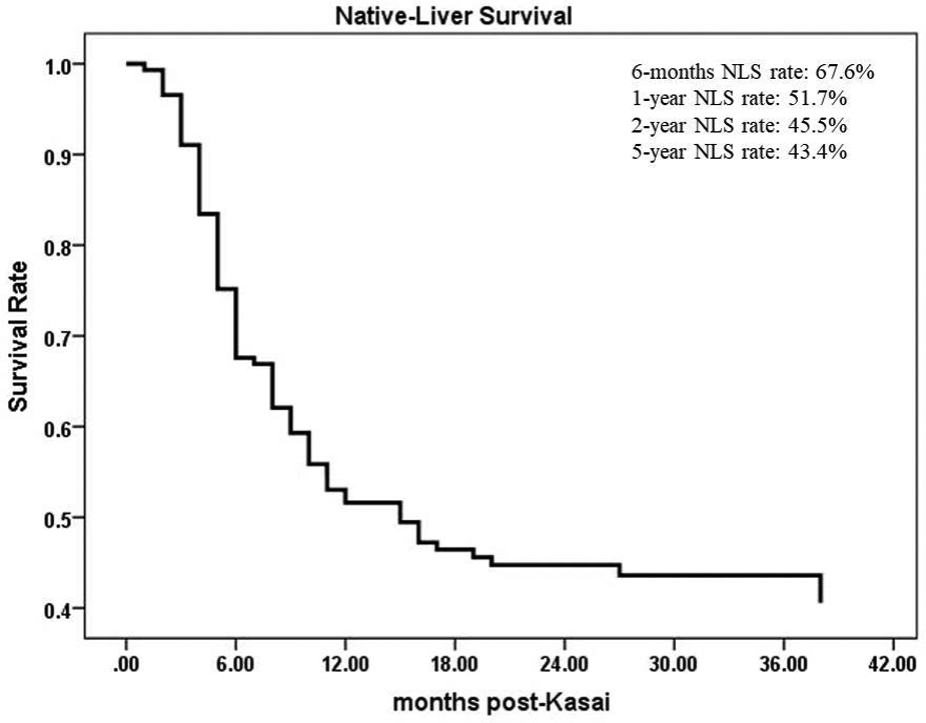
The NLS curve of total patients with BA. NLS, native liver survival.

**Figure 3:**
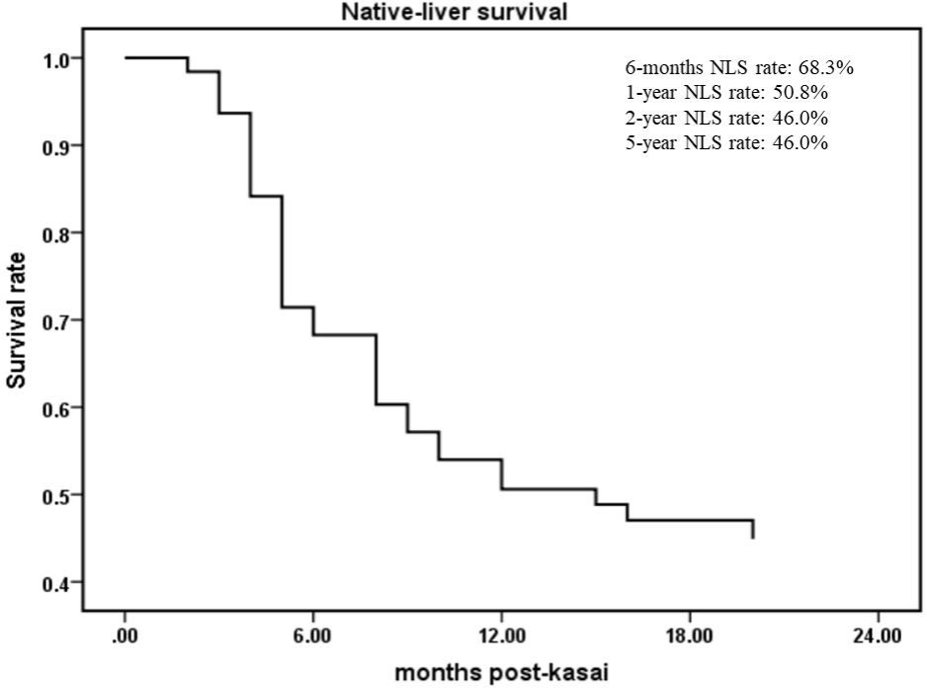
The NLS curve of BA patients without cholangitis after KPE. NLS, native liver survival; KPE, Kasai portoenterostomy.

From June 2016 to December 2021, a total of 413 children were diagnosed with BA by laparoscopic biliary exploration and postoperative pathology, 214 of whom received Kasai portoenterostomy. Sixty-nine children were excluded for the following reasons: (1) Type I and II BA; (2) incomplete case data; and (3) lost to follow-up. Finally, 145 infants were included in the study (Figure 1), including 73 males and 72 females, with an average operative age of 70 ± 16 days. Among these children (*n* = 145), 82 had cholangitis (56.6%, 82/145), of which 40 had early cholangitis (48.8%, 40/82) and 33 had recurrent cholangitis (40.2%, 33/82). The median follow-up period was 29 months (range, 2–75 months). The NLS rates were 67.6%, 51.7%, 45.5% and 43.4% at 6 months, 1 year, 2 years and 5 years following KPE (Figure 2), while the NLS rates for infants without cholangitis after KPE were 68.3%, 50.8%, 46.0% and 46.0% at 6 months, 1 year, 2 years and 5 years (Figure 3), respectively.

### 3.2. Comparison of indexes before KPE among different cholangitis groups

The preoperative γ-GT and TBA levels were significantly higher in the cholangitis (Cho) group than in the noncholangitis (Noncho) group (*P *= 0.034, *P *= 0.014), while other preoperative indexes were not significantly different (Table 1).

**Table 1:** Demographic data, preoperative liver function tests and liver pathology.

	Cho group (*n* = 82)	Noncho group (*n* = 63)	*P*-value	Early cho group (*n* = 40)	Late cho group (*n* = 42)	*P*-value	Occasional cho group (*n* = 49)	Recurrent cho group (*n* = 33)	*P*-value	EBD-G in noncho group (*n* = 21)	EBD-P in noncho group (*n* = 42)	*P*-value	EBD-G group (*n* = 65)	EBD-P group (*n* = 80)	*P*-value
Gender			0.133			0.126			1.000			0.28			0.406
Male (*n*, %)	46 (56.1%)	27 (42.9%)		26 (65%)	20 (47.6%)		27 (55.1%)	19 (57.6%)		7 (33.3%)	20 (47.6%)		30 (46.2%)	43 (53.7%)	
Female (*n*, %)	36 (43.9%)	36 (57.1%)		14 (35%)	22 (52.4%)		22 (44.9%)	14 (42.4%)		14 (66.7%)	22 (52.4%)		35 (53.8%)	37 (46.3%)	
Age at KPE [days]	71.94 ± 17.8	66.87 ± 14.0	0.065	75.15 ± 18.48	68.88 ± 16.79	0.111	71.29 ± 18.97	72.91 ± 16.16	0.688	63.57 ± 13.45	68.52 ± 14.14	0.188	69.17 ± 16.99	70.2 ± 16.0	0.708
Gestational age [weeks]	38.18 ± 1.47	38.33 ± 1.56	0.552	38.35 ± 1.1	38.02 ± 1.75	0.317	38.16 ± 1.14	38.21 ± 1.87	0.883	38.43 ± 1.03	38.29 ± 1.77	0.734	38.4 ± 0.9	38.13 ± 1.85	0.275
Mode of delivery			0.368			0.818			0.158			0.422			0.72
Spontaneous delivery (*n*, %)	53 (64.6%)	46 (73.0%)		25 (62.5%)	28 (66.7%)		35 (71.4%)	18 (54.5%)		14 (66.7%)	32 (76.2%)		43 (66.2%)	56 (70.0%)	
Cesarean section (*n*, %)	29 (35.4%)	17 (27.0%)		15 (37.5%)	14 (33.3%)		14 (28.6%)	15 (45.5%)		7 (33.3%)	10 (23.8%)		22 (33.8%)	24 (30.0%)	
Feeding practice			0.109			0.25			0.767			0.103			0.299
Breast feeding (*n*, %)	17 (20.7%)	22 (34.9%)		6 (15%)	11 (26.2%)		11 (22.4%)	6 (18.2%)		11 (52.4%)	11 (26.2%)		21 (32.3%)	18 (22.5%)	
Mixed feeding (*n*, %)	39 (47.6%)	28 (44.5%)		18 (45%)	21 (50%)		24 (49.0%)	15 (45.5%)		6 (28.6%)	22 (52.4%)		30 (46.2%)	37 (46.3%)	
Milk feeding (*n*, %)	26 (31.7%)	13 (20.6%)		16 (40%)	10 (23.8%)		14 (28.6%)	12 (36.3%)		4 (19.0%)	9 (21.4%)		14 (21.5%)	25 (31.2%)	
Body weight[kg]	5.0 ± 0.58	4.81 ± 0.7	0.076	5.11 ± 0.59	4.9 ± 0.55	0.104	4.99 ± 0.64	5.02 ± 0.48	0.829	4.60 ± 0.69	4.91 ± 0.70	0.101	4.91 ± 0.61	4.93 ± 0.67	0.854
BMI	15.62 ± 1.67	15.77 ± 2.03	0.626	15.49 ± 1.69	15.74 ± 1.67	0.502	15.6 ± 1.36	15.65 ± 2.07	0.913	16.02 ± 2.00	15.65 ± 2.05	0.49	15.71 ± 1.74	15.67 ± 1.91	0.899
Liver Fibrosis			0.791			0.483			0.728			0.442			0.052
F0	2 (2.4%)	3 (4.8%)		1 (2.5%)	1 (2.4%)		2 (4.1%)	0 (0.0%)		0 (0.0%)	3 (7.14%)		0 (0.0%)	5 (6.3%)	
F1	20 (24.4%)	16 (25.4%)		12 (30.0%)	8 (19.0%)		10 (20.4%)	10 (30.3%)		6 (28.6%)	10 (23.8%)		13 (20.0%)	23 (28.7%)	
F2	14 (17.1%)	10 (15.9%)		7 (17.5%)	7 (16.7%)		7 (14.3%)	7 (21.2%)		3 (14.3%)	7 (16.7%)		13 (20.0%)	11 (13.8%)	
F3	41 (50.0%)	29 (46.0%)		16 (40.0%)	25 (59.5%)		29 (59.2%)	12 (36.4%)		9 (42.9%)	20 (47.6%)		32 (49.2%)	38 (47.5%)	
F4	5 (6.1%)	5 (7.9%)		4 (10.0%)	1 (2.4%)		1 (2.0%)	4 (12.1%)		3 (14.2%)	2 (4.76%)		7 (10.8%)	3 (3.7%)	
ALT (U/L)	183.27 ± 133.85	193.97 ± 156.68	0.658	179.9 ± 130.78	186.48 ± 138.21	0.826	201.49 ± 157.62	156.21 ± 82.58	0.094	187.43 ± 168.01	197.24 ± 134.03	0.817	184.98 ± 145.8	190.3 ± 143.01	0.826
AST (U/L)	234.62 ± 135.06	280.4 ± 261.88	0.174	224.5 ± 131.61	244.26 ± 139.17	0.511	246.31 ± 161.17	217.27 ± 81.82	0.287	283.81 ± 187.33	278.69 ± 174.68	0.942	249.25 ± 242.22	258.79 ± 160.93	0.777
γ-GT (U/L)	666.76 ± 485.3	507.35 ± 387.77	**0**.034*	634.8 ± 465.8	601.95 ± 353.15	0.719	646.84 ± 534.94	696.33 ± 406.68	0.653	668.05 ± 419.84	474.62 ± 363.94	0.064	630.22 ± 389.99	545.91 ± 410.46	0.211
TB (*μ*mol/L)	157.14 ± 39.56	167.62 ± 48.42	0.154	151.19 ± 40.63	162.81 ± 38.13	0.185	158.56 ± 41.88	155.03 ± 36.37	0.695	165.91 ± 50.38	168.47 ± 48.01	0.845	160.23 ± 43.14	162.88 ± 44.53	0.719
DB (μmol/L)	123.81 ± 31.43	124.09 ± 33.74	0.96	121.31 ± 34.99	126.2 ± 27.84	0.485	123.02 ± 32.47	124.99 ± 30.28	0.782	126.67 ± 33.02	122.79 ± 34.42	0.671	125.77 ± 33.1	122.44 ± 31.84	0.54
TBA (μmol/L)	172.23 ± 70.51	145.89 ± 52.17	**0**.014*	175.79 ± 68.96	168.85 ± 72.62	0.659	170.78 ± 68.83	174.38 ± 73.96	0.822	137.46 ± 47.70	150.10 ± 54.33	0.369	169.93 ± 76.73	153.36 ± 51.5	0.139
ALB (g/L)	41.57 ± 3.39	40.94 ± 2.94	0.246	42.0 ± 2.99	41.16 ± 3.72	0.261	41.49 ± 3.69	41.69 ± 2.93	0.787	40.96 ± 2.91	40.94 ± 2.99	0.974	41.84 ± 3.1	40.86 ± 3.24	0.065

Cho, cholangitis; Noncho, noncholangitis; EBD-G, early bile drainage-good; EBD-P, early bile drainage-poor; KPE, Kasai portoenterostomy; BMI, body mass index; ALT, alanine transaminase; AST, aspartate transaminase; γ-GT, gammaglutamyl transpeptidase; TB, total bilirubin; DB, direct bilirubin; TBA, total bile acid; Alb, albumin.

* *P* < 0.05.

Bold value means that the result was statistically significant.

### 3.3. Comparison of NLS among different cholangitis groups

**Figure 4:**
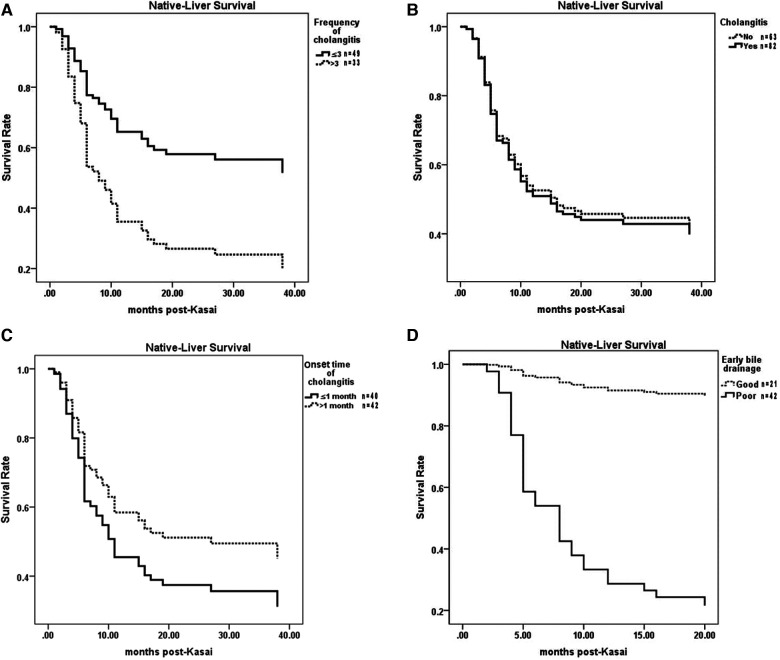
Comparisons of NLS among different cholangitis groups. (**A**) Recurrent cholangitis group vs Occasional cholangitis group. (**B**) Cholangitis group vs Noncholangitis group. (**C**) Early cholangitis group vs. Late cholangitis group. (**D**) EBD-G group vs. EBD-P group. NLS, native liver survival. EBD, early bile drainage.

The NLS rate in cases of recurrent cholangitis was significantly lower than that in cases of occasional cholangitis (*P* = 0.003) (Figure 4A). However, compared with the noncholangitis group, the cholangitis group had no significant difference in the NLS rate (*P* = 0.829) (Figure 4B).

### 3.4. Comparison of NLS between the EBD-G and EBD-P groups

Among children without cholangitis after KPE, the NLS rate in children with EBD-G was significantly higher than that in children with EBD-P (*P *= 0.000) (Figure 4D).

### 3.5. Association of EBD and cholangitis

The EBD was significantly related to the occurrence of cholangitis (*P *= 0.018) (Table 2), which showed that the number of children with cholangitis increased in the EBD-G group. EBD was also significantly related to the frequency of cholangitis (*P *= 0.03) (Table 2), which showed that the number of children without cholangitis increased in the EBD-P group. However, there was no significant correlation between EBD and the onset time of cholangitis (*P *= 0.055) (Table 2).

**Table 2:** Correlation of EBD and cholangitis.

	EBD-G group (*n* = 65)	EBD-P group (*n* = 80)	*χ^2^*	*P*
Occurrence of cholangitis				
Yes	44 (67.7%)	38 (47.5%)	5.951	0.018*
None	21 (32.3%)	42 (52.5%)		
Onset time of cholangitis				
Early	20 (30.8%)	18 (22.5%)	5.781	0.055
Late	23 (35.4%)	19 (23.8%)		
None	22 (33.8%)	43 (53.7%)		
Frequency of cholangitis				
None	21 (32.3%)	42 (52.5%)	7.029	0.03*
Occasional	24 (36.9%)	25 (31.3%)		
Recurrent	20 (30.8%)	13(16.2%)		

EBD-G, early bile drainage-good; EBD-P, early bile drainage-poor.

* *P* < 0.05.

Bold value means that the result was statistically significant.

## 4. Discussion

BA is still a leading cause of liver transplantation in children, although great progress has been made in the diagnosis and treatment of BA in recent years (15, 16). Approximately 50% of affected infants need LT during the first two years of life because of severe complications, such as malnutrition, portal hypertension and coagulopathy. In Europe, the native liver survival was 41%–55% at 5 years following Kasai portoenterostomy (17), which was similar to our results.

Cholangitis not only seriously affects the quality of life of BA children after KPE but also places great economic and psychological burdens on their parents (13). The effect of cholangitis on NLS is controversial. Compared with the cholangitis group, the nonicteric native liver survival (nNLS) rate was significantly higher in children without cholangitis and with jaundice clearance within 6 months after KPE (6). Conversely, Khayat et al. (9) found that cholangitis was significantly associated with longer survival. Our study found that there was no significant difference in the NLS rate between the Cho group and the Noncho group; this was consistent with the results of previous studies (18, 19).

Cholangitis may be related to microbiota migration and the immune inflammatory response. Gut microbiota is also closely related to feeding practice and delivery modes (20, 21). Recently, Zheng et al. (3) reported that the gut microbiota in infants with cholangitis was significantly different from that in infants without cholangitis. The results of this study suggest that there are no significant differences in feeding practice and delivery modes among different subgroups of cholangitis, avoiding the possibility of these factors indirectly participating in the occurrence of cholangitis by affecting the composition of gut microbiota. Serum γ-GT, which is produced mostly by the liver and the bile duct, is sensitive for detecting cholangitis and can be used as a marker of disease progression. The γ-GT level increased even if the liver was slightly insulted (22). Harpavat et al. (23) reported that bile acid levels can be used as a useful prognostic biomarker for infants achieving normalized bilirubin levels after KPE. This study found that compared with the Noncho group, the levels of preoperative γ-GT and total bile acid (TBA) in the Cho group were increased significantly, indicating that bile duct injury and poor bile drainage existed in children with BA before KPE. When bacteria located in the Roux loop invade the bile duct, they are more likely to cause cholangitis. This was consistent with the results of previous studies (6, 24). The research on the relationship between γ-GT and prognosis, as well as the relationship between TBA and prognosis, will be conducted in future work.

The frequency and onset time of cholangitis were significantly related to postoperative quality of life (24). Ramachandran et al. (25) showed that increased episodes of postoperative cholangitis were a predictor of poor outcome after KPE. Additionally, repeated episodes of postoperative cholangitis seem to be associated with a worse outcome for BA patients (26, 27). Calinescu et al. (7) found that the greater the occurrence of cholangitis, the lower the NLS rate. This may be because repeated attacks of cholangitis can aggravate the progression of liver fibrosis and cirrhosis, thereby reducing the NLS rate. Liu et al. (6) also found that if early cholangitis was not treated in time, it easily progressed to recurrent cholangitis, thereby reducing the NLS rate. The results of this study support the above findings, and the NLS rate of children with recurrent cholangitis was significantly lower than that of children with occasional cholangitis. Early cholangitis can decrease the clearance of jaundice and decrease the NLS (6). However, the results of this study suggest that there was no significant difference between the early cholangitis group and the late cholangitis group, which may be related to the treatment effect of cholangitis. Timely and effective treatment of cholangitis will slow the progression to recurrent cholangitis, thus improving the NLS rate. In contrast, if early cholangitis cannot be treated in a timely and effective manner and progresses to recurrent cholangitis, then the NLS rate will be reduced.

Children without cholangitis have totally different NLS according to the different EBDs 1 month after KPE. In our study, we found that only the EBD-G group had a good NLS rate. In contrast, in the EBD-P group, the NLS rate was significantly reduced. Serum bilirubin rapidly decreases to very low levels after KPE, which can prolong the NLS time by delaying the process of liver fibrosis (28). Sundaram et al. (29) also reported that the 10-year NLS rate of children with jaundice cleared 3 months after KPE ranged from 75%-90%, while that of children with persistent jaundice at 3 years after KPE was only 20%. This may be related to the effect of bile drainage after KPE. For children without cholangitis with good bile drainage after KPE, jaundice subsided quickly, and the NLS rate increased. Conversely, for children without cholangitis and without effective bile drainage, the obstruction of bile drainage and lack of jaundice clearance may lead to a shortened NLS time. Therefore, for children without cholangitis after KPE, clinicians should pay careful attention to the effect of early bile drainage.

Bile drainage was closely related to the occurrence and frequency of cholangitis. The incidence of cholangitis was significantly higher in children who cleared jaundice than in those who did not (25). Good bile drainage and jaundice clearance were associated with a lower risk of cholangitis. In this study, we found that early bile drainage was significantly related to the occurrence and frequency of cholangitis but not to the onset time of cholangitis. Most of the children with good early bile drainage had cholangitis; however, the number of children without cholangitis increased significantly among children with poor early bile drainage. This serves as a reminder that bile drainage is a necessary condition for the occurrence of cholangitis. Without adequate bile drainage, even if the occurrence of cholangitis is less frequent, the NLS time will be shortened.

This study is limited by several factors. First, our study only included 145 children with BA, and all of them were Type III and came from one center. Second, although this study found that the effect of early biliary drainage significantly affected the NLS rate in children without cholangitis after KPE, this result still needs to be verified in large-sample and multicenter studies. Finally, whether the effect of early bile drainage can be given different scores according to clinical manifestations and laboratory test results as an indicator to predict long-term NLS requires more detailed work in the future.

## 5. Conclusions

Recurrent cholangitis was an important factor affecting NLS after KPE. For children who did not have cholangitis after KPE, only early bile drainage was better and the NLS time was longer, thereby providing a theoretical basis for clinical practice.

## Data Availability

The original contributions presented in the study are included in the article/**Supplementary Material**, further inquiries can be directed to the corresponding author.
