# Supplementary material for: Early bile drainage improves native liver survival in biliary atresia without cholangitis
Source: Front Pediatr. 2023 Jul 12;11:1189792. doi: 10.3389/fped.2023.1189792 (PMC10368976; doi:10.3389/fped.2023.1189792)
Supplement: Supplementary file 1 [file Table1.pdf]

Supplementary Table 1. Details for NLS after KPE

	NLS rate after KPE			
	6-months	1-year	2-year	5-year
BA cases (n=145)	67.6%(98/145)	51.7%(75/145)	45.5%(66/145)	43.4%(63/145)
BA cases without cholangitis (n=63)	68.3%(43/63)	50.8%(32/63)	46.0%(29/63)	46.0%(29/63)

BA, biliary atresia. NLS, native liver survival. KPE, Kasai portoenterostomy.
